# Aberrant DNA methylation and expression of SPDEF and FOXA2 in airway epithelium of patients with COPD

**DOI:** 10.1186/s13148-017-0341-7

**Published:** 2017-04-24

**Authors:** J. Song, I. H. Heijink, L. E. M. Kistemaker, M. Reinders-Luinge, W. Kooistra, J. A. Noordhoek, R. Gosens, C. A. Brandsma, W. Timens, P. S. Hiemstra, M. G. Rots, M. N. Hylkema

**Affiliations:** 1 Department of Pathology and Medical Biology, University Medical Center Groningen, University of Groningen, Groningen, The Netherlands; 2 GRIAC Research Institute, University Medical Center Groningen, University of Groningen, Groningen, The Netherlands; 3 Department of Biochemistry and Molecular Biology, School of Basic Medical Sciences, Tianjin Medical University, Tianjin, China; 4 Department of Immunology, School of Basic Medical Sciences, Tianjin Medical University, Tianjin, China; 5 Department of Molecular Pharmacology, University of Groningen, Groningen, The Netherlands; 6 Department of Pulmonology, Leiden University Medical Center, Leiden, The Netherlands; 7 Department of Pathology and Medical Biology EA10, University Medical Center Groningen, Hanzeplein 1, 9713 GZ Groningen, The Netherlands

**Keywords:** SPDEF, FOXA2, DNA methylation, Mucus, COPD

## Abstract

**Background:**

Goblet cell metaplasia, a common feature of chronic obstructive pulmonary disease (COPD), is associated with mucus hypersecretion which contributes to the morbidity and mortality among patients. Transcription factors SAM-pointed domain-containing Ets-like factor (SPDEF) and forkhead box protein A2 (FOXA2) regulate goblet cell differentiation. This study aimed to (1) investigate DNA methylation and expression of *SPDEF* and *FOXA2* during goblet cell differentiation and (2) compare this in airway epithelial cells from patients with COPD and controls during mucociliary differentiation.

**Methods:**

To assess DNA methylation and expression of *SPDEF* and *FOXA2* during goblet cell differentiation, primary airway epithelial cells, isolated from trachea (non-COPD controls) and bronchial tissue (patients with COPD), were differentiated by culture at the air-liquid interface (ALI) in the presence of cytokine interleukin (IL)-13 to promote goblet cell differentiation.

**Results:**

We found that *SPDEF* expression was induced during goblet cell differentiation, while *FOXA2* expression was decreased. Importantly, CpG number 8 in the *SPDEF* promoter was hypermethylated upon differentiation, whereas DNA methylation of *FOXA2* promoter was not changed. In the absence of IL-13, COPD-derived ALI-cultured cells displayed higher *SPDEF* expression than control-derived ALI cultures, whereas no difference was found for *FOXA2* expression. This was accompanied with hypomethylation of CpG number 6 in the *SPDEF* promoter and also hypomethylation of CpG numbers 10 and 11 in the *FOXA2* promoter.

**Conclusions:**

These findings suggest that aberrant DNA methylation of *SPDEF* and *FOXA2* is one of the factors underlying mucus hypersecretion in COPD, opening new avenues for epigenetic-based inhibition of mucus hypersecretion.

**Electronic supplementary material:**

The online version of this article (doi:10.1186/s13148-017-0341-7) contains supplementary material, which is available to authorized users.

## Background

Chronic bronchitis, one of the clinical phenotypes of chronic obstructive pulmonary disease (COPD), is characterized by goblet cell metaplasia and excessive mucus production and secretion, which contributes to the morbidity and mortality of patients [1–3]. The tracheobronchial epithelium of the human airways consists of basal cells, ciliated cells, club (Clara) cells, goblet cells, and neuroendocrine cells [4, 5]. Basal cells serve as the progenitor cells from which goblet cells and ciliated cells are derived, both in the normal airway epithelia renewal process and during abnormal remodeling in disease [6, 7]. Goblet cell differentiation is dictated by a large network of genes, in which transcription factors SAM-pointed domain-containing ETS-like factor (SPDEF) and forkhead box protein A2 (FOXA2) are two key regulators. SPDEF is required for goblet cell differentiation and mucus production, including the major secreted airway mucin MUC5AC (mucin 5AC) [8–10], whereas FOXA2 is a potent inhibitor of goblet cell differentiation in the lung [11–13]. Recent studies have shown that *SPDEF* is expressed higher (messenger RNA (mRNA)) in the large airway epithelium of smokers compared to non-smokers [14, 15] and also expressed higher (protein) in lung tissue of patients with asthma and COPD compared to healthy controls [16]. FOXA2 was shown to be reduced (both mRNA and protein) and was negatively correlated with MUC5AC in bronchial epithelium of patients with asthma [17] and in nasal tissue of individuals with chronic rhinosinusitis [18]. In addition, *FOXA2* was expressed lower (mRNA) in small airway epithelium of both healthy smokers and COPD smokers compared to non-smokers [19].

DNA methylation is an important mechanism in the regulation of gene expression during adult stem cell renewal and differentiation, as is shown for the differentiation of hematopoietic, epidermal, and intestinal stem cells [20–23]. However, the role of DNA methylation in airway basal cell differentiation has not been evaluated. Other studies showed that aberrant DNA methylation was associated with dysregulation of *SPDEF* and *FOXA2* expression in lung cancer [24, 25], although DNA methylation regulation of *SPDEF* and *FOXA2* expression has not been assessed in lung tissue of patients with COPD.

In this study, we aimed to investigate the DNA methylation and expression of *SPDEF* and *FOXA2* during goblet cell differentiation and further identify whether DNA methylation and expression of *SPDEF* and *FOXA2* are different in patients with COPD compared to control subjects after in vitro airway epithelial cell differentiation. Expression of *SPDEF* target genes *MUC5AC* and anterior gradient 2 (*AGR2*) and the ciliated cell related gene Forkhead Box J1 (*FOXJ1*) were additionally assessed.

## Methods

### Culture of PBEC cells

Primary human bronchial epithelial cells (PBECs) were obtained from bronchial tissue harvested from transplant recipient lungs of 16 patients with GOLD (Global Initiative for Chronic Obstructive Lung Disease) stage IV COPD and residual tracheal and main stem bronchial tissue from 17 transplant donors (non-COPD controls). No information was available from the transplant donors. Selection criteria for transplant donors are listed in the Eurotransplant guidelines including the absence of primary lung disease, such as asthma and COPD, and no more than 20 pack years of smoking history. Characteristics of patients with COPD and details on the experimental design are shown in Table 1.

**Table 1:** Characteristics of subjects and experimental design

Culture condition		Age (years)	Sex	Smoking status	Pack years	FEV1%pred	FEV1/FVC%	*N* (RNA)	*N* (DNA)
ALI with IL-13^a^	COPD 1	56	M	Ex	30	31	29	5	5
	COPD 2	59	F	Ex	41	87	59		
	COPD 3	60	F	Ex	20	33	38		
	COPD 4	56	F	Ex	30	14	25		
	COPD 5	61	F	Ex	26	21	28		
	Non-COPD controls 1–6	NA	NA	NA	NA	NA	NA	6	6
ALI without IL-13^b^	COPD 6	58	F	Ex	40	18	25	7	8
	COPD 7	61	F	Ex	35	20	25		
	COPD 8	55	F	Ex	18	19	52		
	COPD 9	49	M	Ex	11	20	22		
	COPD 10	60	M	Ex	38	16	29		
	COPD 11	48	M	Ex	25	12	23		
	COPD 12	58	F	Ex	38	60	46		
	COPD 13	63	M	Non	0	41	45		
	COPD 14	53	M	Ex	30	25	25		
	COPD 15	57	M	Ex	30	11	31		
	COPD 16	57	F	Ex	45	23	24		
	Non-COPD controls 11–17	NA	NA	NA	NA	NA	NA	7	7

*Abbreviations*: *COPD* chronic obstructive pulmonary disease, *M* male, *F* female, *Ex* ex-smoker, *Non* non-smoker, *NA* not available, *FEV1%pred* forced expiratory volume during the first second as percentage of predicted, *FEV1/FVC%* the ratio of FEV1 to FVC (forced vital capacity), *ALI* air-liquid interface culture, *IL-13* interleukin 13, *N (RNA)* number of RNA samples in total, *N (DNA)* number of DNA samples in total

^a^Cells were cultured at ALI in the presence of IL-13 and harvested after 0, 14, 21 and 28 days

^b^Cells were cultured at ALI without IL-13 and harvested after 14 days

**Fig. 1:**
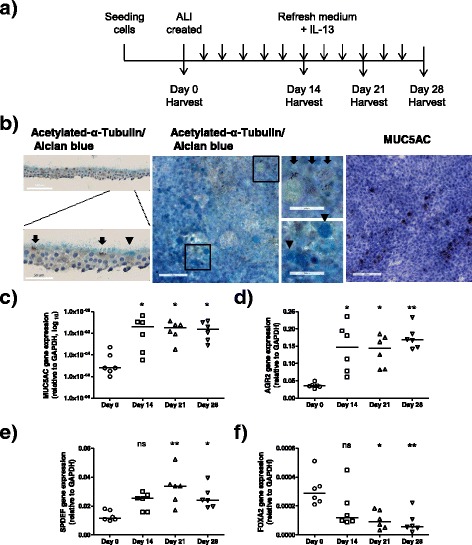
Characterization of the differentiation state of primary bronchial epithelial cells (PBEC) cultured in the air-liquid interface (ALI) model. **a** Schematic representation of the ALI culture model. PBECs were seeded on to a transwell insert and grown until confluence. Thereafter, apical medium was removed to create an ALI. Cells were harvested after 0, 14, 21, or 28 days for RNA, DNA, and morphology analysis. **b**–**d** PBEC from control subjects 1–6 (Table 1, *n* = 6) were cultured at ALI with IL-13 stimulation. **b** Representative images of immunohistochemistry staining on the differentiated ciliated cells and goblet cells at ALI day 21. Ciliated cells were determined by acetylated-α-tubulin antibody staining and specified by arrows in the images; goblet cells were determined by Alcian Blue staining and MUC5AC antibody staining and were specified by arrow heads in the images. mRNA expressions of **c** *MUC5AC*, **d** *AGR2*, **e** *SPDEF*, and **f** *FOXA2* were analyzed by real-time quantitative PCR at four different time points. Medians are indicated. Significance was tested by the Kruskal-Wallis non-parametric test with Dunn’s posttest data. *ns* not significant. *p < 0.05; **p < 0.01. Data from days 14, 21, and 28 were compared to day 0

For the initial experiments, the third passage PBECs from control subjects 1–6 and patients with COPD 1–5 (Table 1) were cultured in bronchial epithelium growth medium (BEGM, Lonza, Walkersville, MD, USA) until confluence on fibronectin/collagen pre-coated transwell inserts (0.4-μm pore size, 12-mm diameter; Corning, NY, USA) and were allowed to differentiate at air-liquid interface (ALI) culture in the presence of interleukin (IL)-13 (1 ng/ml; Peprotech, Rocky Hill, NJ, USA) to enhance goblet cell differentiation (Fig. 1a) as previously described [26]. Cells were harvested after 0, 14, 21, or 28 days of air exposure for analysis of morphology, mRNA expression, and DNA methylation. For the latter experiments, the third passage PBECs from control subjects 7–17 and COPD patients 6–16 (Table 1) were cultured in BEGM medium until confluence on pre-coated transwell inserts (0.4-μm pore size, 6.5-mm diameter; Corning) and allowed to differentiate at ALI culture without IL-13 (Fig. 4a) as described previously [27]. The cells were harvested after 14 days of air exposure for analysis of mRNA expression and DNA methylation.

For morphology analyses in transverse, the transwell inserts were formalin-fixed and embedded in paraffin according to Corning’s instructions, and cross sections (5 μm thick) were analyzed after immunohistochemistry staining. For morphology analyses in horizontal, transwell inserts were fixed with 4% (*w*/*v*) paraformaldehyde (Merck, Darmstadt, Germany), after which the membrane was cut into four quarters and analyzed after immunohistochemistry staining.

### Immunohistochemistry

Paraffin sections and insert membranes were investigated for the presence of ciliated cells and goblet cells using standard immunohistochemical procedures. Ciliated cells were determined by staining slides with a monoclonal mouse anti-acetylated α-tubulin antibody (Sigma-Aldrich T7451, St. Louis, MO, USA) and visualized with diaminobenzidine (DAB, Sigma) solution. In the same sections, goblet cells were subsequently visualized after staining with Alcian Blue. MUC5AC-positive cells were determined with a monoclonal mouse anti-MUC5AC antibody (Abcam, ab3649, Cambridge, UK) and visualized with 3-amino-9-ethylcarbazole (AEC, Sigma).

### mRNA expression by quantitative real-time PCR

Total RNA from PBEC was extracted using Trizol reagent (Thermo Fisher Scientific, Carlshad, USA), according to the manufacturer’s instructions. RNA quantity and purity were assessed using NanoDrop 2000 (Thermo Scientific). Then, 500 ng of total RNA was used for complementary DNA (cDNA) synthesis with random primers using Superscript II RNase H - Reverse transcriptase (Thermo Fisher Scientific). *SPDEF*, *MUC5AC*, *AGR2*, *FOXA2*, *FOXJ1*, and *GAPDH* expression were quantified using 5 ng cDNA, qPCRMasterMix Plus (Eurogentec, Belgium), and Taqman gene-specific primer/probes (*SPDEF*: Hs01026050_m1; *MUC5AC*: Hs00873651_Mh; *AGR2*: Hs00356521_m1; *FOXA2*: Hs00232764_m1; *FOXJ1*: Hs00230964_m1; *GAPDH*: Hs02758991_g1, Thermo Fisher Scientific) for 40 cycles with LightCycler® 480 Real-Time PCR System (Roche, Basel, Switzerland). Data were analyzed with LightCycler® 480 SW 1.5 software (Roche) and the Fit point method, according to the manufacturer’s instructions. Expression levels relative to *GAPDH* were determined with the formula 2^−ΔCp^ (Cp means crossing points). Samples for which no amplification could be detected were assigned a Cp value of 40 (the total number of PCR cycles).

### Methylation analysis by pyrosequencing

For DNA methylation analysis of the target regions, genomic DNA was extracted with chloroform-isopropanol and bisulfite converted using the EZ DNA Methylation-Kit (Zymo Research), following the manufacturer’s protocol. Bisulfite-converted DNA (10–20 ng) was amplified by PCR in a 25 μl reaction using the Pyromark PCR kit (Qiagen). Pyrosequencing was performed on the Pyromark Q24 pyrosequencer (Qiagen) according to the manufacturer’s guidelines, using a specific sequencing primer. Analysis of methylation levels at each CpG site was determined using Pyromark Q24 Software (Qiagen). The pyrosequencing primers’ information is presented in Additional file 1: Table S1.

### Statistics

Results obtained from qRT-PCR and pyrosequencing are expressed as median and range, respectively. For comparisons between undifferentiated (day 0) and differentiated epithelial cells (days 14, 21, and 28), the Kruskal-Wallis non-parametric test with Dunn’s posttest was applied. For comparisons of expression levels between COPD and controls, the Mann-Whitney *U* test was applied. Correlation analyses of the level of methylation level and mRNA within the same sample was tested by Spearman non-parametric correlation test. All statistical analyses were performed with Prism v5.0.

## Results

### *SPDEF* and *FOXA2* expression profiles during epithelial cell differentiation in the presence of IL-13

First, in order to validate the role of transcription factors *SPDEF* and *FOXA2* in goblet cell differentiation, PBECs from six control individuals (Table 1, control subjects 1–6) were ALI cultured for 28 days in the presence of IL-13 to promote goblet cell differentiation (Fig. 1a). At days 0, 14, 21, and 28, the differentiation state of PBEC was characterized by immunohistochemistry staining and quantitative real-time PCR. As expected, this protocol induced the differentiation of goblet cells as shown by the Alcian Blue-positive cells and MUC5AC-positive cells after 14 to 28 days of ALI culture (Fig. 1b). Immunohistochemistry staining demonstrated that approximately 5% of the cells represented goblet cells, whereas the majority of cells consisted of ciliated cells (tubulin positive) or other cells (negative for both Alcian Blue and tubulin).

Goblet cell differentiation was accompanied by increased expression of *MUC5AC* (59.6-fold) and Anterior gradient 2 (*AGR2*) (4.5-fold), which encodes a potential chaperone required for mucin packaging (Fig. 1c, d). These changes were observed after 14 days of ALI culture compared to expression at day 0, and the expression levels remained consistent after 21 and 28 days of ALI culture (Fig. 1c, d). As expected, increased *MUC5AC* expression was accompanied by increased expression of transcription factor *SPDEF* (2.8-fold at day 21, 2.2-fold at day 28) and decreased expression of *FOXA2* (to 28.6% of the start level at day 21, to 19.7% at day 28) (Fig. 1e, f). In line with the differentiation of ciliated cells, increased expression of the ciliated cell marker Forkhead Box J1 (*FOXJ1*, a key transcription factor for ciliated cell differentiation) was also found (Additional file 2: Figure S1).

### DNA methylation dynamics within *SPDEF* and *FOXA2* promoter during IL-13-induced goblet cell differentiation

**Fig. 2:**
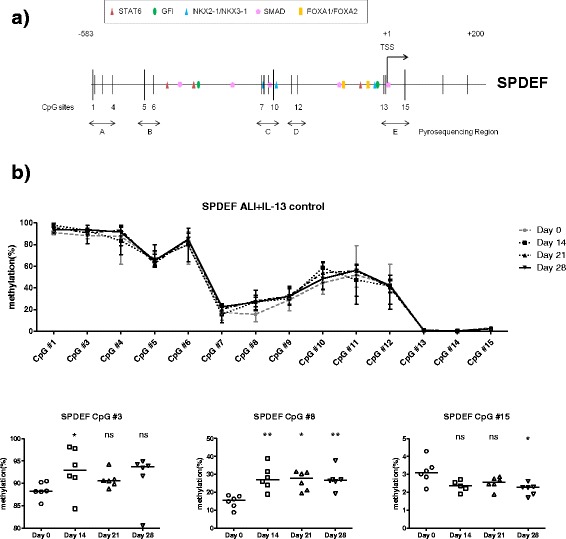
Dynamic changes of DNA methylation in the *SPDEF* promoter during goblet cell differentiation of PBEC from control subjects. **a** Schematic representation of the promoter region of the *SPDEF* gene, outlining the putative binding sites for the most relevant transcription factors (STAT6, GFI, NKX2-1/NKX3-1, SMAD, and FOXA1/FOXA2) as analyzed using MatInspector software. The transcription start site was shown as +1. CpGs are indicated as *vertical bars*. DNA methylation status of 15 CpGs was analyzed using pyrosequencing for the indicated areas. **b** PBEC from control subjects1–6 (Table 1, *n* = 6) were cultured at ALI, and DNA methylation levels were analyzed at four different time points. Data represent the connected median methylation levels (min to max) of different CpG sites at each time points, and differential methylated CpG sites are specified with medians indicated. Significance was tested by the Kruskal-Wallis non-parametric test with Dunn’s posttest data. *ns* not significant. *p < 0.05; **p < 0.01. Data from days 14, 21, and 28 were compared to day 0

Next, DNA methylation of *SPDEF* and *FOXA2* was assessed in the total cell population at different time points during goblet cell differentiation (Table 1, control subjects 1–6). As DNA methylation of CpG sites in the region of the first exon and the promoter (including the TSS) has been described to be tightly correlated with the gene transcription [28], DNA methylation of CpG sites in these specific regions were analyzed in the following experiments. Fourteen CpG sites within the *SPDEF* promoter and first exon were analyzed at five loci (Fig. 2a, loci A to E) using pyrosequencing analysis. Compared to day 0 of ALI culture, methylation levels of CpG site numbers 3 and 8 increased significantly at day 14 (CpG number 3, from 88 to 93%; CpG number 8, from 16 to 27%) and the methylation level of CpG number 8 remained higher after 21 and 28 days of ALI culture (Fig. 2b). Importantly, the methylation level in CpG number 8 was positively correlated with mRNA expression of *SPDEF* (Spearman *r* = 0.567, *p* < 0.004, Additional file 3: Figure S2). CpG number 15 was the only CpG site of which methylation was decreased at day 28 of ALI culture compared to day 0 (from very low level of 3% at day 0 to 2% at day 28, Fig. 2b).

**Fig. 3:**
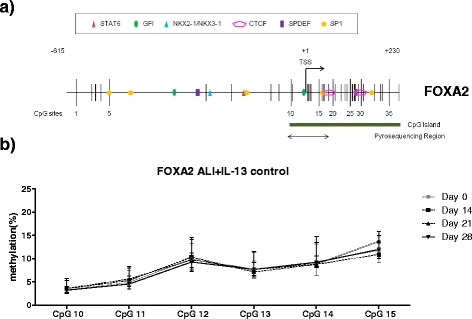
Dynamic changes of DNA methylation in the *FOXA2* promoter during goblet cell differentiation of PBEC from control subjects. **a** Schematic representations of the promoter region of the *FOXA2* gene, outlining the putative binding sites for transcription factors (STAT6, GFI, NKX2-1/NKX3-1, CTCF, SPDEF, and SP1) (MatInspector, top relevants). DNA methylation levels of six CpG sites in the promoter, which were part of a CpG island (indicated in the figure), were examined for the indicated areas. **b** PBEC from control subjects 1–6 (Table 1, *n* = 6) were cultured at ALI and DNA methylation levels were analyzed at four different time points. Data represent the connected median methylation levels (min to max) of different CpG sites at each time points

For *FOXA2*, six CpG sites that were part of a CpG island in the promoter and first exon were examined (Fig. 3a, CpG numbers 10–15). Methylation in these sites did not change despite of the observed *FOXA2* downregulation during differentiation (Fig. 3b).

In order to investigate goblet cell differentiation in COPD, primary cells from five patients with COPD (Table 1, COPD patients 1–5) were cultured at ALI in the presence of IL-13 using the same protocol. Similar to the observations in cells from controls, increased expression of the goblet cell markers (*MUC5AC*, *AGR2*, and *SPDEF*) and the ciliated cell marker (*FOXJ1*) were observed after 14 to 28 days of ALI culture (Additional file 4: Figure S3a). In addition, a same trend of DNA methylation dynamics in the *SPDEF* promoter was observed (Additional file 5: Figure S4a) and the methylation level of CpG number 8 was positively correlated with mRNA expression of *SPDEF* (Spearman *r* = 0.6165, *p* < 0.004, Additional file 5: Figure S4b). However, different from the observations in control, *FOXA2* expression did not decreased during the IL-13-induced goblet cell differentiation of cells from patients with COPD (Additional file 4: Figure S3b), whereas loss of DNA methylation in the *FOXA2* promoter was observed (CpG number 14, from 12% at day 0 to 8% at day 28; CpG number 15, from 15% at day 0 to 11% at day 28, Additional file 6: Figure S5).

### Aberrant expression and DNA methylation of *SPDEF* and *FOXA2* in promotor region airway epithelial cells of COPD patients

**Fig. 4:**
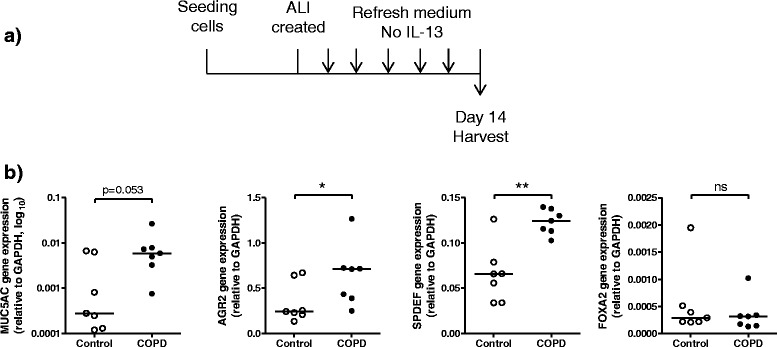
Differential mRNA expression profiles in PBECs from COPD patients and control subjects. **a** Schematic representation of the ALI culture model. PBECs from control 7–17 and COPD 6–16 (Table 1) were seeded onto transwell inserts and grown to confluence. Thereafter, apical medium was removed to create an ALI. Cells were harvested after 14 days for RNA and DNA analysis. **b** Expressions of *MUC5AC*, *AGR2*, *SPDEF*, and *FOXA2* were analyzed by real-time quantitative PCR. Significance was tested by the Mann-Whitney *U* test. Medians are indicated. *ns* not significant. *p < 0.05; **p < 0.01

In order to investigate putative differences in airway epithelial cell differentiation between COPD and control, PBECs from COPD and control subjects (Table 1, COPD patients 6–16 and control subjects 7–17) were differentiated at ALI culture *in the absence of IL-13*, and gene expression and DNA methylation levels were compared in the total cell population after 14 days of ALI culture (Fig. 4a). To exclude variables such as batch effects, cultures and analyses were performed simultaneously using the same reagents. We found that epithelial cells derived from patients with COPD had significantly higher mRNA expressions of *MUC5AC*, *AGR2*, and *SPDEF* than epithelial cells from controls (*MUC5AC*, 21.1-fold; *SPDEF*, 1.9-fold; *AGR2*, 2.9-fold, Fig. 4b), whereas no difference was found in *FOXA2* and *FOXJ1* expression between COPD-derived cells and control-derived cells (Fig. 4b and Additional file 7: Figure S6).

**Fig. 5:**
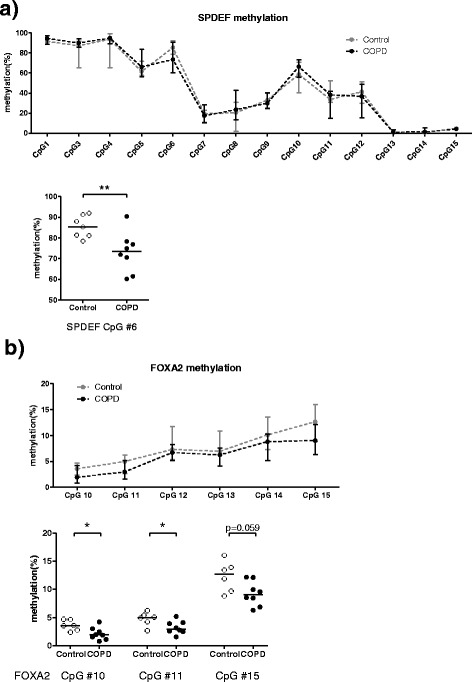
Differential DNA methylation of *SPDEF* and *FOXA2* in the PBECs from COPD patients and control subjects. PBECs from control 7–17 and COPD 6–16 (Table 1) were differentiated at ALI for 14 days, and DNA methylation of **a** *SPDEF* and **b** *FOXA2* was analyzed by pyrosequencing. Data represent the connected median methylation levels (min to max) of different CpG sites in controls or in COPD, and differential methylated CpG sites between COPD-derived cultures and control-derived cultures are shown. Significance was tested by the Mann-Whitney *U* test. Medians are indicated. *ns* not significant. *p < 0.05; **p < 0.01

COPD-derived cells presented significant lower methylation levels of CpG site number 6 in the *SPDEF* promoter (85% in control, 73% in COPD, Fig. 5a) and also lower methylation levels of three CpG sites in the *FOXA2* promoter (CpG number 10: 4% in control and 2% in COPD, CpG number 11: 5% in control and 3% in COPD, CpG number 15: 13% in control and 9% in COPD, Fig. 5b) than control-derived cells at ALI day 14.

## Discussion

In this study, we had two main observations. First, *SPDEF* expression in epithelial cells was increased during IL-13-induced goblet cell differentiation and correlated with altered DNA methylation in epithelial cells, derived from both controls and patients with COPD. Second, *SPDEF* and *MUC5AC* were higher expressed in ALI-differentiated epithelial cells from patients with COPD compared to controls, which was accompanied with DNA hypomethylation in the *SPDEF* promoter. Furthermore, *FOXA2* expression was not affected during airway epithelial cell differentiation in patients with COPD but was decreased during differentiation in controls. This was not accompanied by changes in DNA methylation.

SPDEF has been described, by us and others, to be upregulated in human bronchial epithelial cells in response to IL-13 [26, 29, 30]. Our study confirms the increased *SPDEF* expression after IL-13 stimulation, both in epithelial cells from controls and patients with COPD. During this process, *SPDEF* upregulation was accompanied by dynamic changes of methylation at several CpG sites of the *SPDEF* promoter. Importantly, methylation of CpG number 8 was consistently and positively correlated to SPDEF expression in the IL-13-induced goblet cell differentiation of cells from both controls and patients with COPD. This is of interest as CpG number 8 locates in a putative binding site for transcription factor NK2 homeobox 1 (NKX2-1). NKX2-1 is an airway epithelial-specific transcription factor which has been found to inhibit SPDEF expression and prevent ovalbumin-induced goblet cell differentiation and lung inflammation in transgenic *Nkx2-1* overexpressing mice [31]. Interestingly, NKX2-1 was found to be methylation sensitive in the regulation of its target gene surfactant protein B and myosin-binding protein H [32, 33]. The hypermethylation of CpG number 8 in the *SPDEF* promoter might prevent binding of NKX2-1, leading to increased transcription of *SPDEF* during differentiation. We speculate that NKX2-1 is a transcription inhibitor of *SPDEF* and that DNA methylation impairs the NKX2-1 inhibitory effect on the *SPDEF* promoter activity. For now, there is no direct evidence showing that *SPDEF* is a target gene of NKX2-1 except for their inverse role in goblet cell differentiation and mucus production. It will be interesting to further investigate the relation of NKX2-1 binding and *SPDEF* promoter methylation (particularly CpG number 8) to *SPDEF* promoter activity in the future.

*SPDEF* and *MUC5AC* have previously been shown to be highly expressed in bronchial epithelium of patients with COPD [16], which is in agreement with our findings, in which increased expression of *SPDEF*, *MUC5AC*, and *AGR2* was found in COPD-derived ALI cultures when compared to controls. Moreover, our data demonstrate that there was hypomethylation of CpG number 6 in the *SPDEF* promoter in the COPD-derived ALI cultures, which is in line with the over-expression and hypomethylation of *SPDEF* in lung cancer [24]. There are some confounding factors that may have contributed to the difference we found between COPD and non-COPD controls. Although no genetic background information of non-COPD controls or COPD cases were available, there is no evidence showing that the reported CpG sites include any underlying SNPs except CpG site number 2 in the SPDEF promoter, which was excluded because of a known G/A SNP. Besides lack of information on medication, no information exists on whether the controls have been smoking. However, we do not assign our findings to a possible current smoke effect in non-COPD controls as Beane et al. have shown that expression of SPDEF and MUC5AC was significantly higher, instead of lower, in current smokers compared to former and never smokers [14]. In addition, the location of sampling of the primary cells from control and COPD was not exactly the same. Epithelial cells from COPD were obtained from the large bronchus, whereas samples from transplant donor controls were obtained from the trachea/main stem area. However, goblet cell density is decreased from proximal to distal airways [34, 35], and distal airways have been shown to be less prone for goblet cell metaplasia by diminished expression of IL-13 signaling components, including IL-13 receptor IL-13Rα1, SPDEF, and FOXA3 [36]. These considerations support our conclusion that patients with COPD have an increased expression of *SPDEF* and *MUC5AC* compared to controls and suggest that our results might even be an underestimation.

As expected, *FOXA2*, a known repressor of goblet cell differentiation [11, 13, 17, 29, 37] was reduced during IL-13-induced epithelial cell differentiation. As *FOXA2* is negatively regulated by SPDEF [9, 38] and two putative binding sites for SPDEF locate in the promoter and first exon of *FOXA2*, SPDEF may decrease *FOXA2* expression directly. *FOXA2* is also regulated by promoter DNA methylation. *FOXA2* was shown to be repressed and hypermethylated in its promoter in lung cancer [25], whereas it was also shown that *FOXA2* was activated with hypermethylation in the promoter during endoderm development [39]. In our study, we observed that *FOXA2* was hypomethylated in cells from patients with COPD after goblet cell differentiation not only in the presence of IL-13 (CpG numbers 14 and 15) but also in the absence of IL-13 (CpG numbers 10, 11, and 15). In both cases, hypomethylation of *FOXA2* was not accompanied with a change in expression level. These phenomena could be explained by the minor methylation differences (around 2 to 4%), which might not result in any biological effect on transcription, or a decreased binding of repressive transcription factors/increased binding of active transcription factors.

It is of note that FOXA2 is also essential for proper establishment of cellular junctions and maintenance of polarity [40], whereas *FOXA2*, together with other apical junctional complex-related genes, was shown to be decreased (mRNA) in small airway epithelium of both healthy smokers and COPD smokers compared to non-smokers [19]. In agreement with previous findings, cultured bronchial epithelial cells from patients with COPD displayed abnormalities with reduced capacity to form epithelial junctions and regenerate a mucociliary epithelium [41], which might be driven by aberrant DNA methylation profiles. It is therefore important to validate our finding and further investigate the biological role of DNA methylation in the *FOXA2* promoter during airway epithelial cell differentiation and also in COPD in the future.

We assessed DNA methylation in the total population of differentiated cells, of which the goblet cell population represents about 5%. Using pure cell populations of basal cells, ciliated cells, club (Clara) cells, and goblet cells in future studies will increase the opportunity to find more differentiated methylation loci/regions. However, even using the total cell population, our approach turned out to be sensitive enough to reveal difference between COPD and controls, which may be indicative for the magnitude of this methylation difference.

## Conclusions

In summary, we have demonstrated that *SPDEF* expression is increased during IL-13-induced goblet cell differentiation, which correlates to hypermethylation of CpG number 8 in the *SPDEF* promoter. *SPDEF* expression is also higher in COPD-derived ALI cultures compared to control-derived ALI cultures in the absence of IL-13, which is accompanied with hypomethylation of CpG number 6 in the *SPDEF* promoter. Moreover, *FOXA2* is hypomethylated (CpG numbers 14 and 15) during IL-13-induced goblet cell differentiation and also hypomethylated (CpG numbers 10 and 11) in COPD without a change in expression level. This shows the complex biology of airway epithelial cell differentiation where different transcription factors are involved and expression and DNA methylation mutually affect each other. Our study has shown the potential relevance of *SPDEF* regarding mucus hypersecretion in COPD and the involvement of altered methylation patterns in this phenomenon, and these insights might prove useful for the future development of epigenetic-based anti-mucus therapeutic strategies [42].

## Additional files

Additional file 1: Table S1. Primers locations and sequences.

Additional file 2: Figure S1. *FOXJ1* mRNA expression in primary bronchial epithelial cells (PBEC) after air-liquid interface (ALI) culture for 14, 21, and 28 days.

Additional file 3: Figure S2. Correlation between methylation level of CpG number 8 in the *SPDEF* promoter and *SPDEF* mRNA level during goblet cell differentiation of PBECs from control subjects.

Additional file 4: Figure S3. Characterization of differentiated PBECs isolated from patients with COPD and cultured in the air-liquid interface (ALI) model for 14, 21, and 28 days.

Additional file 5: Figure S4. Dynamic changes of DNA methylation in the *SPDEF* promoter during goblet cell differentiation of PBECs from patients with COPD.

Additional file 6: Figure S5. Dynamic changes of DNA methylation in the *FOXA2* promoter during goblet cell differentiation of PBECs from patients with COPD.

Additional file 7: Figure S6. Differential mRNA expression of *FOXJ1* in the PBECs from COPD patients and control subjects.

## References

[1] Miravitlles M (2011). Cough and sputum production as risk factors for poor outcomes in patients with COPD. Respir Med.

[2] Allinson JP, Hardy R, Donaldson GC, Shaheen SO, Kuh D, Wedzicha JA (2016). The presence of chronic mucus hypersecretion across adult life in relation to chronic obstructive pulmonary disease development. Am J Respir Crit Care Med.

[3] Ramos FL, Krahnke JS, Kim V (2014). Clinical issues of mucus accumulation in COPD. Int J Chron Obstruct Pulmon Dis.

[4] Herriges M, Morrisey EE (2014). Lung development: orchestrating the generation and regeneration of a complex organ. Development.

[5] Crystal RG (2014). Airway basal cells. The “smoking gun” of chronic obstructive pulmonary disease. Am J Respir Crit Care Med.

[6] Hogan BL, Barkauskas CE, Chapman HA, Epstein JA, Jain R, Hsia CC, Niklason L, Calle E, Le A, Randell SH (2014). Repair and regeneration of the respiratory system: complexity, plasticity, and mechanisms of lung stem cell function. Cell Stem Cell.

[7] Rock JR, Randell SH, Hogan BLM (2010). Airway basal stem cells: a perspective on their roles in epithelial homeostasis and remodeling. Dis Model Mech.

[8] Park KS, Korfhagen TR, Bruno MD, Kitzmiller JA, Wan H, Wert SE, Khurana Hershey GK, Chen G, Whitsett JA (2007). SPDEF regulates goblet cell hyperplasia in the airway epithelium. J Clin Invest.

[9] Chen G, Korfhagen TR, Xu Y, Kitzmiller J, Wert SE, Maeda Y, Gregorieff A, Clevers H, Whitsett JA (2009). SPDEF is required for mouse pulmonary goblet cell differentiation and regulates a network of genes associated with mucus production. J Clin Investig.

[10] Rajavelu P, Chen G, Xu Y, Kitzmiller JA, Korfhagen TR, Whitsett JA (2015). Airway epithelial SPDEF integrates goblet cell differentiation and pulmonary Th2 inflammation. J Clin Invest.

[11] Wan H, Kaestner KH, Ang SL, Ikegami M, Finkelman FD, Stahlman MT, Fulkerson PC, Rothenberg ME, Whitsett JA (2004). Foxa2 regulates alveolarization and goblet cell hyperplasia. Development.

[12] Chen G, Wan H, Luo F, Zhang L, Xu Y, Lewkowich I, Wills-Karp M, Whitsett JA (2010). Foxa2 programs Th2 cell-mediated innate immunity in the developing lung. J Immunol.

[13] Tang X, Liu XJ, Tian C, Su Q, Lei Y, Wu Q, He Y, Whitsett JA, Luo F (2013). Foxa2 regulates leukotrienes to inhibit Th2-mediated pulmonary inflammation. Am J Respir Cell Mol Biol.

[14] Beane J, Sebastiani P, Liu G, Brody JS, Lenburg ME, Spira A (2007). Reversible and permanent effects of tobacco smoke exposure on airway epithelial gene expression. Genome Biol.

[15] Sridhar S, Schembri F, Zeskind J, Shah V, Gustafson AM, Steiling K, Liu G, Dumas YM, Zhang X, Brody JS (2008). Smoking-induced gene expression changes in the bronchial airway are reflected in nasal and buccal epithelium. BMC Genomics.

[16] Chen G, Korfhagen TR, Karp CL, Impey S, Xu Y, Randell SH, Kitzmiller J, Maeda Y, Haitchi HM, Sridharan A (2014). Foxa3 induces goblet cell metaplasia and inhibits innate antiviral immunity. Am J Respir Crit Care Med.

[17] Park SW, Verhaeghe C, Nguyenvu LT, Barbeau R, Eisley CJ, Nakagami Y, Huang X, Woodruff PG, Fahy JV, Erle DJ (2009). Distinct roles of FOXA2 and FOXA3 in allergic airway disease and asthma. Am J Respir Crit Care Med.

[18] Luo Q, Zhang J, Wang H, Chen F, Luo X, Miao B, Wu X, Ma R, Luo X, Xu G (2015). Expression and regulation of transcription factor FoxA2 in chronic rhinosinusitis with and without nasal polyps. Allergy, Asthma Immunol Res.

[19] Shaykhiev R, Otaki F, Bonsu P, Dang DT, Teater M, Strulovici-Barel Y, Salit J, Harvey BG, Crystal RG (2011). Cigarette smoking reprograms apical junctional complex molecular architecture in the human airway epithelium in vivo. Cell Mol Life Sci.

[20] Bock C, Beerman I, Lien WH, Smith ZD, Gu H, Boyle P, Gnirke A, Fuchs E, Rossi DJ, Meissner A (2012). DNA methylation dynamics during in vivo differentiation of blood and skin stem cells. Mol Cell.

[21] Berdasco M, Esteller M (2011). DNA methylation in stem cell renewal and multipotency. Stem Cell Res Ther.

[22] Zhang X, Ulm A, Somineni HK, Oh S, Weirauch MT, Zhang HX, Chen X, Lehn MA, Janssen EM, Ji H (2014). DNA methylation dynamics during ex vivo differentiation and maturation of human dendritic cells. Epigenetics Chromatin.

[23] Sheaffer KL, Kim R, Aoki R, Elliott EN, Schug J, Burger L, Schübeler D, Kaestner KH (2014). DNA methylation is required for the control of stem cell differentiation in the small intestine. Genes Dev.

[24] Selamat SA, Chung BS, Girard L, Zhang W, Zhang Y, Campan M, Siegmund KD, Koss MN, Hagen JA, Lam WL (2012). Genome-scale analysis of DNA methylation in lung adenocarcinoma and integration with mRNA expression. Genome Res.

[25] Basseres DS, D’Alò F, Yeap BY, Löwenberg EC, Gonzalez DA, Yasuda H, Dayaram T, Kocher ON, Godleski JJ, Richards WG (2012). Frequent downregulation of the transcription factor Foxa2 in lung cancer through epigenetic silencing. Lung Cancer (Amsterdam, Netherlands).

[26] Kistemaker LE, Hiemstra PS, Bos IS, Bouwman S, van den Berge M, Hylkema MN, Meurs H, Kerstjens HA, Gosens R (2015). Tiotropium attenuates IL-13-induced goblet cell metaplasia of human airway epithelial cells. Thorax.

[27] Heijink IH, Postma DS, Noordhoek JA, Broekema M, Kapus A (2010). House dust mite-promoted epithelial-to-mesenchymal transition in human bronchial epithelium. Am J Respir Cell Mol Biol.

[28] Brenet F, Moh M, Funk P, Feierstein E, Viale AJ, Socci ND, Scandura JM (2011). DNA methylation of the first exon is tightly linked to transcriptional silencing. PLoS ONE.

[29] Mertens TC, Hiemstra PS, Taube C (2016). Azithromycin differentially affects the IL-13-induced expression profile in human bronchial epithelial cells. Pulm Pharmacol Ther.

[30] Bai J, Miao B, Wu X, Luo X, Ma R, Zhang J, Li L, Shi J, Li H (2015). Enhanced expression of SAM-pointed domain-containing Ets-like factor in chronic rhinosinusitis with nasal polyps. Laryngoscope.

[31] Maeda Y, Chen G, Xu Y, Haitchi HM, Du L, Keiser AR, Howarth PH, Davies DE, Holgate ST, Whitsett JA (2011). Airway epithelial transcription factor NK2 homeobox 1 inhibits mucous cell metaplasia and Th2 inflammation. Am J Respir Crit Care Med.

[32] Hosono Y, Yamaguchi T, Mizutani E, Yanagisawa K, Arima C, Tomida S, Shimada Y, Hiraoka M, Kato S, Yokoi K (2012). MYBPH, a transcriptional target of TTF-1, inhibits ROCK1, and reduces cell motility and metastasis. EMBO J.

[33] Cao Y, Vo T, Millien G, Tagne JB, Kotton D, Mason RJ, Williams MC, Ramirez MI (2010). Epigenetic mechanisms modulate thyroid transcription factor 1-mediated transcription of the surfactant protein B gene. J Biol Chem.

[34] Cerkez V, Tos M, Mygind N (1986). Quantitative study of goblet cells in the upper lobe of the normal human lung. Arch Otolaryngol Head Neck Surg.

[35] Cerkez V, Tos M, Mygind N (1986). Goblet-cell density in the human-lung—whole-mount study of the normal left lower lobe. Anat Anz.

[36] Vock C, Yildirim AO, Wagner C, Schlick S, Lunding LP, Lee CG, Elias JA, Fehrenbach H, Wegmann M (2015). Distal airways are protected from goblet cell metaplasia by diminished expression of IL-13 signalling components. Clin Exp Allergy.

[37] Hao YH, Kuang ZZ, Walling BE, Bhatia S, Sivaguru M, Chen Y, Gaskins HR, Lau GW (2012). Pseudomonas aeruginosa pyocyanin causes airway goblet cell hyperplasia and metaplasia and mucus hypersecretion by inactivating the transcriptional factor FoxA2. Cell Microbiol.

[38] Yu HM, Li Q, Kolosov VP, Perelman JM, Zhou XD (2010). Interleukin-13 induces mucin 5AC production involving STAT6/SPDEF in human airway epithelial cells. Cell Commun Adhes.

[39] Halpern KB, Vana T, Walker MD (2014). Paradoxical role of DNA methylation in activation of FoxA2 gene expression during endoderm development. J Biol Chem.

[40] Burtscher I, Lickert H (2009). Foxa2 regulates polarity and epithelialization in the endoderm germ layer of the mouse embryo. Development.

[41] Heijink IH, Noordhoek JA, Timens W, van Oosterhout AJM, Postma DS (2014). Abnormalities in airway epithelial junction formation in chronic obstructive pulmonary disease. Am J Respir Crit Care Med.

[42] Song J, Cano-Rodriquez D, Winkle M, Gjaltema RAF, Goubert D, Jurkowski TP, Heijink IH, Rots MG, Hylkema MN (2017). Targeted epigenetic editing of SPDEF reduces mucus production in lung epithelial cells. Am J Physiol Lung Cell Mol Physiol.

