# Supplementary material for: Aberrant DNA methylation and expression of SPDEF and FOXA2 in airway epithelium of patients with COPD
Source: Clin Epigenetics. 2017 Apr 24;9:42. doi: 10.1186/s13148-017-0341-7 (PMC5404321; doi:10.1186/s13148-017-0341-7)
Supplement: Supplementary file 1 — Primers locations and sequences. [file 13148_2017_341_MOESM1_ESM.docx]

Table S1: Primers locations and sequences

| Primer Name | Sequence (5'- 3') | Application |
| --- | --- | --- |
| SPDEF Pyro-A F | GGGTTATGGGAGAGTAAGTTGT | PCR and sequencing for SPDEF-A pyrosequencing |
| SPDEF Pyro-A R | [Biotin]TCTATACCCCACAAAATCCTCAT |  |
| SPDEF Pyro-A Seq | GTTGTTGGTTGGTTT |  |
| SPDEF Pyro-B/C F | GGATTTTGTGGGGTATAGAGAA | PCR and sequencing for SPDEF-B/C pyrosequencing |
| SPDEF Pyro-B/C R | [Biotin]ATTACTACATAACCACTCAACTCATATT |  |
| SPDEF Pyro-B Seq | GGGGTATAGAGAATATAGTT |  |
| SPDEF Pyro-C Seq | TTTAGAATTTTAGTTTTGGATTTA |  |
| SPDEF Pyro-D/E F | ATGAGTTGAGTGGTTATGTAGTAAT | PCR and sequencing for SPDEF-D/E pyrosequencing |
| SPDEF Pyro-D/E R | [Biotin]CCAACCCAAAACTACCTACTAAC |  |
| SPDEF Pyro-D Seq | AGTGGTTATGTAGTAATTAATG |  |
| SPDEF Pyro-E Seq | AATTAGGTTTTGGTTAATTT |  |
| FOXA2 Pyro-F | GTGGGTATTTAGGTTGTGATTGAAAAG | PCR and sequencing for FOXA2 pyrosequencing |
| FOXA2 Pyro-R | ACCCCTCCCTATTACAATTCA |  |
| FOXA2 PyroSeq | GTTGTGATTGAAAAGTAATTTTG |  |
