# Supplementary material for: Aberrant DNA methylation and expression of SPDEF and FOXA2 in airway epithelium of patients with COPD
Source: Clin Epigenetics. 2017 Apr 24;9:42. doi: 10.1186/s13148-017-0341-7 (PMC5404321; doi:10.1186/s13148-017-0341-7)
Supplement: Supplementary file 2 — FOXJ1 mRNA expression in primary bronchial epithelial cells (PBEC) after air-liquid interface (ALI) culture for 14, 21, and 28 days. [file 13148_2017_341_MOESM2_ESM.pptx]

## Slide 1
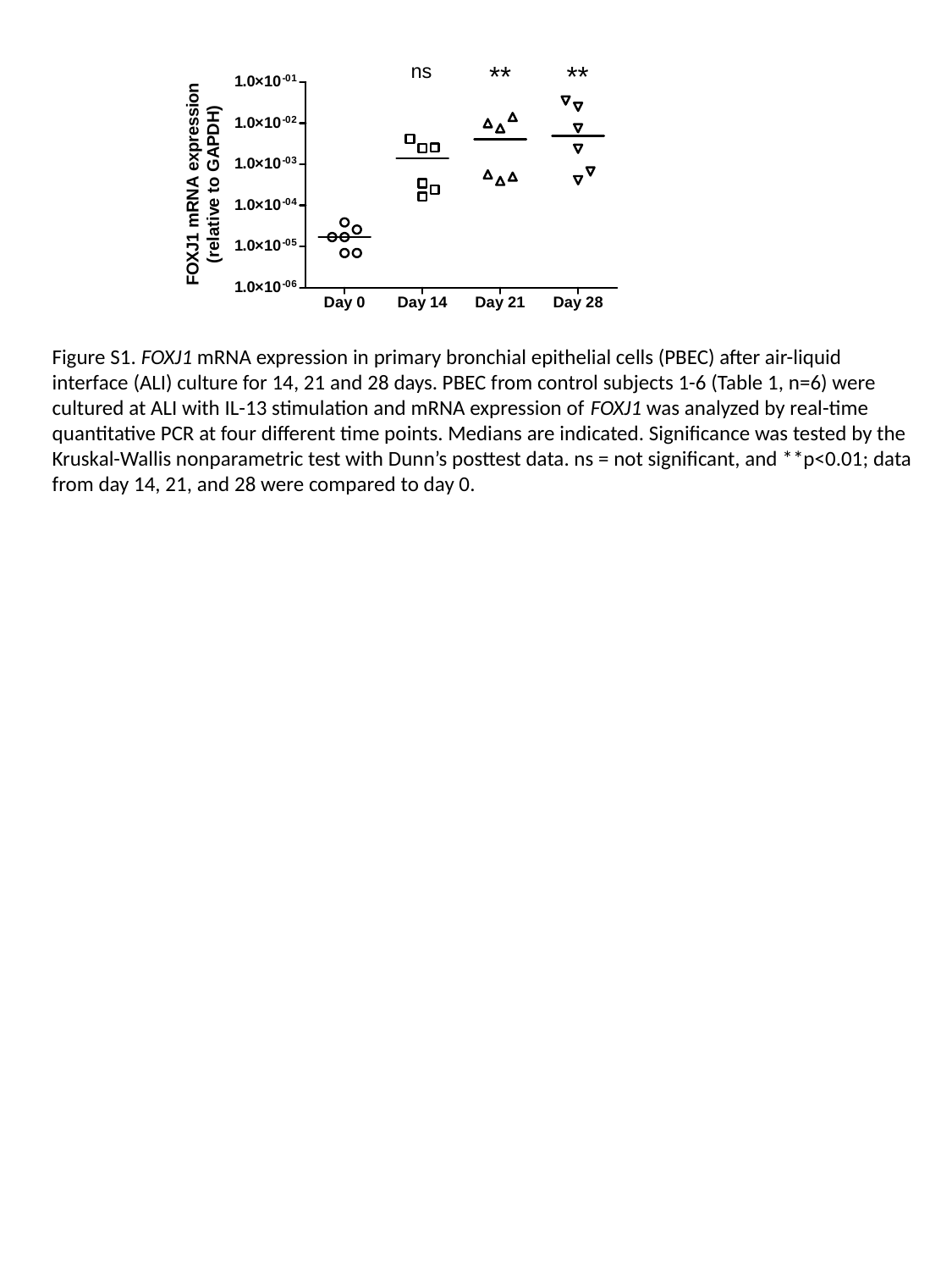

Figure S1. FOXJ1 mRNA expression in primary bronchial epithelial cells (PBEC) after air-liquid interface (ALI) culture for 14, 21 and 28 days. PBEC from control subjects 1-6 (Table 1, n=6) were cultured at ALI with IL-13 stimulation and mRNA expression of FOXJ1 was analyzed by real-time quantitative PCR at four different time points. Medians are indicated. Significance was tested by the Kruskal-Wallis nonparametric test with Dunn’s posttest data. ns = not significant, and **p<0.01; data from day 14, 21, and 28 were compared to day 0.
