# Supplementary material for: Aberrant DNA methylation and expression of SPDEF and FOXA2 in airway epithelium of patients with COPD
Source: Clin Epigenetics. 2017 Apr 24;9:42. doi: 10.1186/s13148-017-0341-7 (PMC5404321; doi:10.1186/s13148-017-0341-7)
Supplement: Supplementary file 3 — Correlation between methylation level of CpG number 8 in the SPDEF promoter and SPDEF mRNA level during goblet cell differentiation of PBECs from control subjects. [file 13148_2017_341_MOESM3_ESM.pptx]

## Slide 1
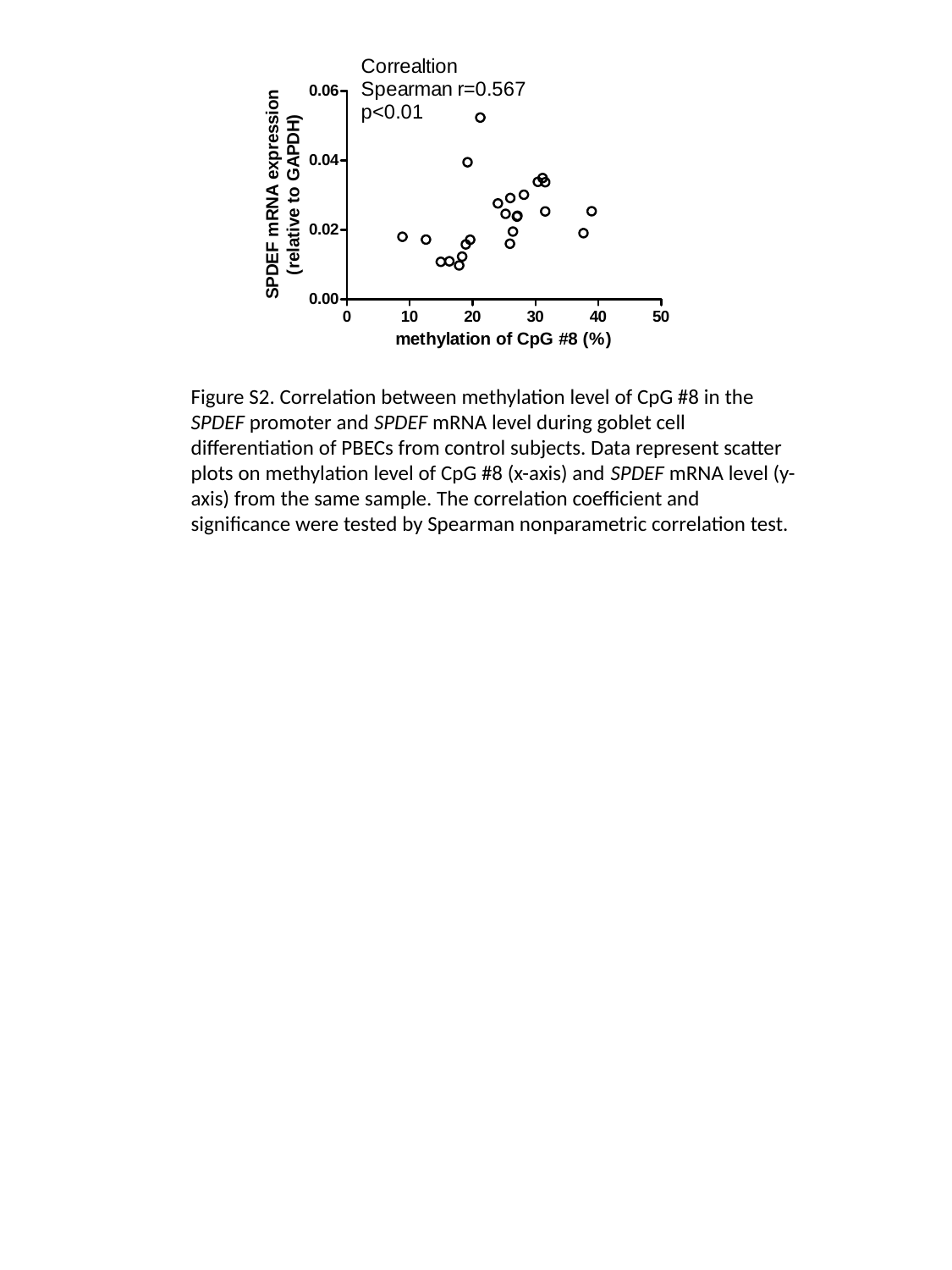

Figure S2. Correlation between methylation level of CpG #8 in the SPDEF promoter and SPDEF mRNA level during goblet cell differentiation of PBECs from control subjects. Data represent scatter plots on methylation level of CpG #8 (x-axis) and SPDEF mRNA level (y-axis) from the same sample. The correlation coefficient and significance were tested by Spearman nonparametric correlation test.
