# Supplementary material for: Aberrant DNA methylation and expression of SPDEF and FOXA2 in airway epithelium of patients with COPD
Source: Clin Epigenetics. 2017 Apr 24;9:42. doi: 10.1186/s13148-017-0341-7 (PMC5404321; doi:10.1186/s13148-017-0341-7)
Supplement: Supplementary file 4 — Characterization of differentiated PBECs isolated from patients with COPD and cultured in the air-liquid interface (ALI) model for 14, 21, and 28 days. [file 13148_2017_341_MOESM4_ESM.pptx]

## Slide 1
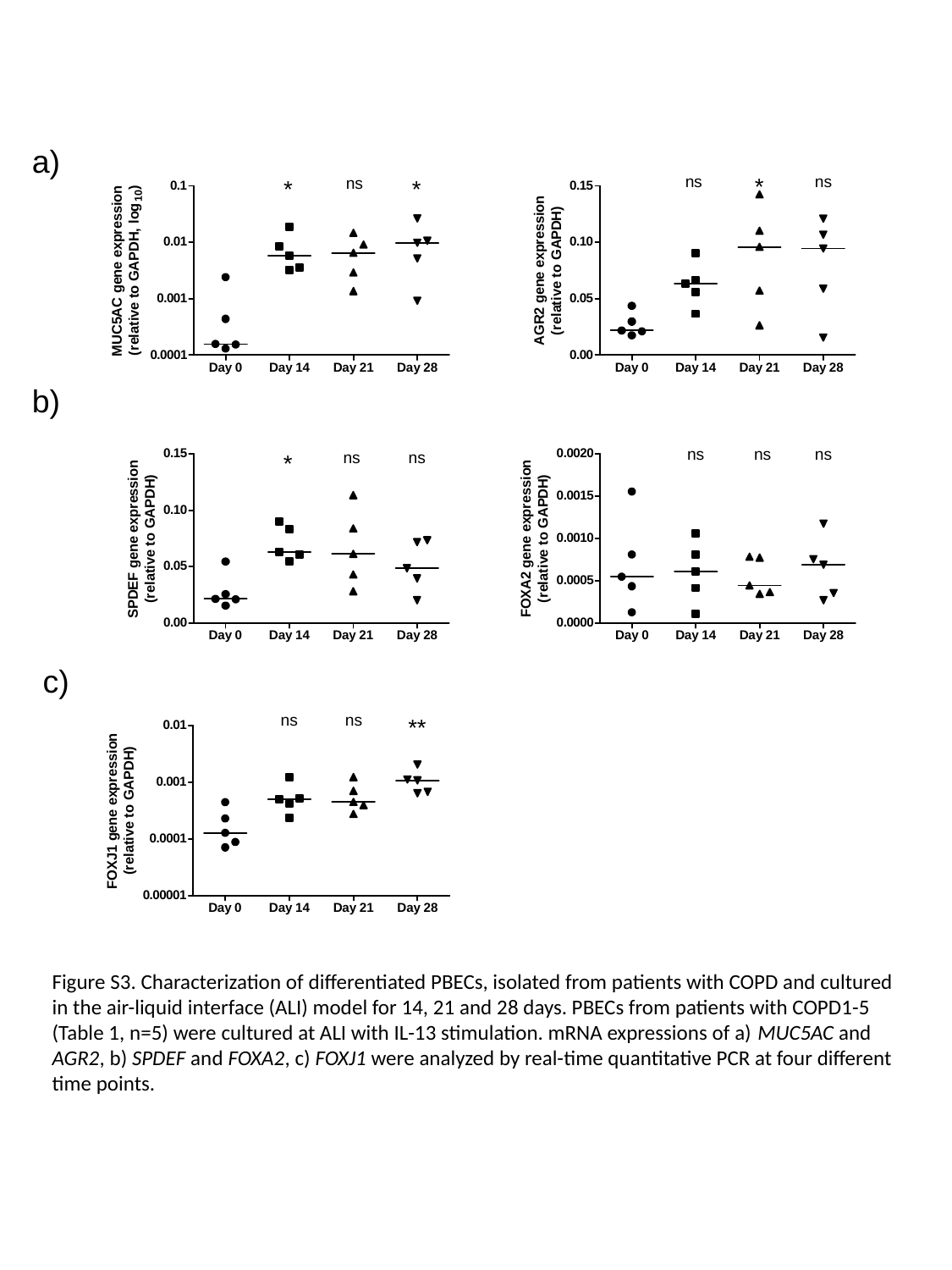

a)
b)
c)
Figure S3. Characterization of differentiated PBECs, isolated from patients with COPD and cultured in the air-liquid interface (ALI) model for 14, 21 and 28 days. PBECs from patients with COPD1-5 (Table 1, n=5) were cultured at ALI with IL-13 stimulation. mRNA expressions of a) MUC5AC and AGR2, b) SPDEF and FOXA2, c) FOXJ1 were analyzed by real-time quantitative PCR at four different time points.
