# Supplementary material for: Aberrant DNA methylation and expression of SPDEF and FOXA2 in airway epithelium of patients with COPD
Source: Clin Epigenetics. 2017 Apr 24;9:42. doi: 10.1186/s13148-017-0341-7 (PMC5404321; doi:10.1186/s13148-017-0341-7)
Supplement: Supplementary file 6 — Dynamic changes of DNA methylation in the FOXA2 promoter during goblet cell differentiation of PBECs from patients with COPD. [file 13148_2017_341_MOESM6_ESM.pptx]

## Slide 1
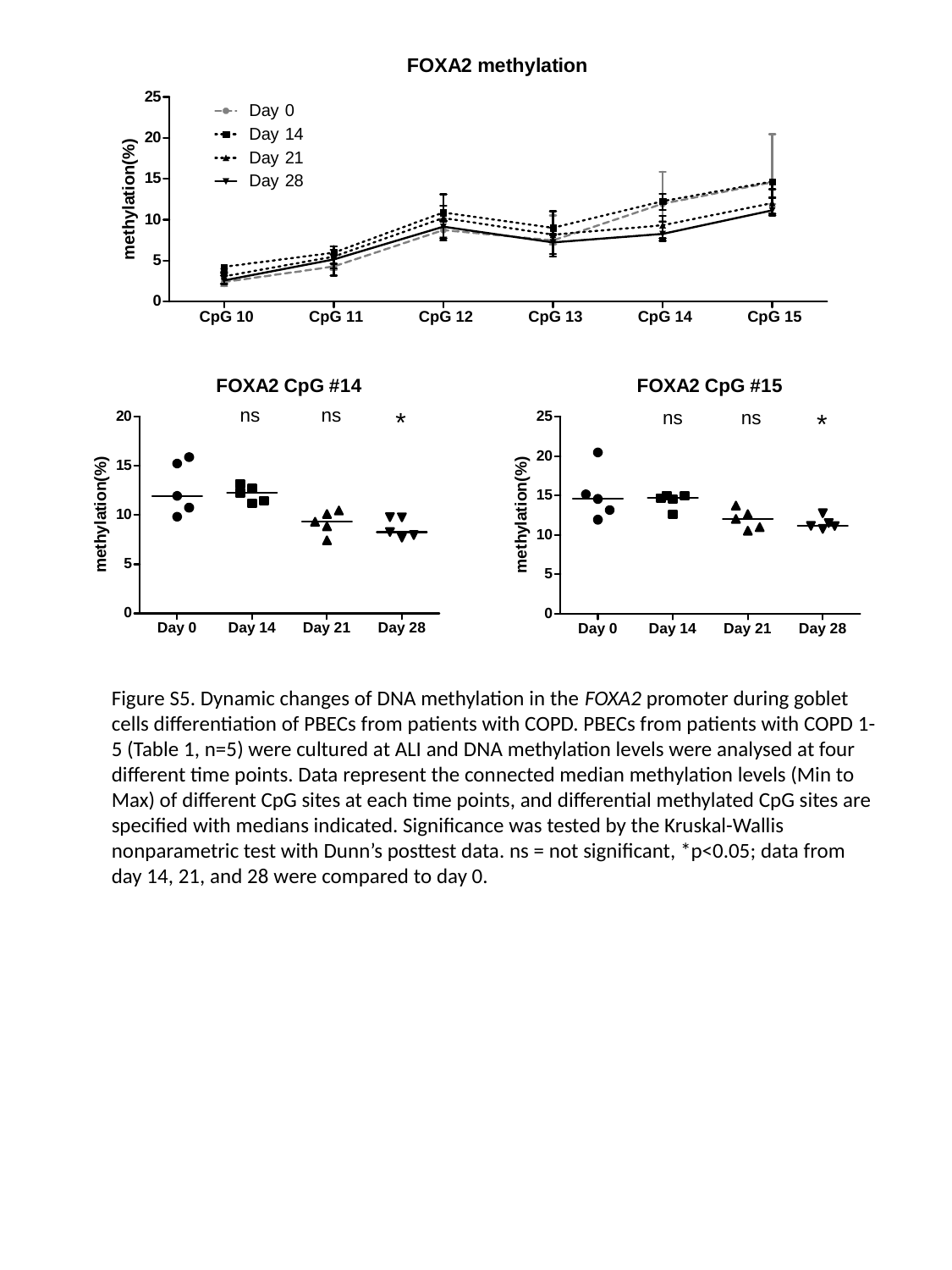

Figure S5. Dynamic changes of DNA methylation in the FOXA2 promoter during goblet cells differentiation of PBECs from patients with COPD. PBECs from patients with COPD 1-5 (Table 1, n=5) were cultured at ALI and DNA methylation levels were analysed at four different time points. Data represent the connected median methylation levels (Min to Max) of different CpG sites at each time points, and differential methylated CpG sites are specified with medians indicated. Significance was tested by the Kruskal-Wallis nonparametric test with Dunn’s posttest data. ns = not significant, *p<0.05; data from day 14, 21, and 28 were compared to day 0.
