# Supplementary material for: Aberrant DNA methylation and expression of SPDEF and FOXA2 in airway epithelium of patients with COPD
Source: Clin Epigenetics. 2017 Apr 24;9:42. doi: 10.1186/s13148-017-0341-7 (PMC5404321; doi:10.1186/s13148-017-0341-7)
Supplement: Supplementary file 7 — Differential mRNA expression of FOXJ1 in the PBECs from COPD patients and control subjects. [file 13148_2017_341_MOESM7_ESM.pptx]

## Slide 1
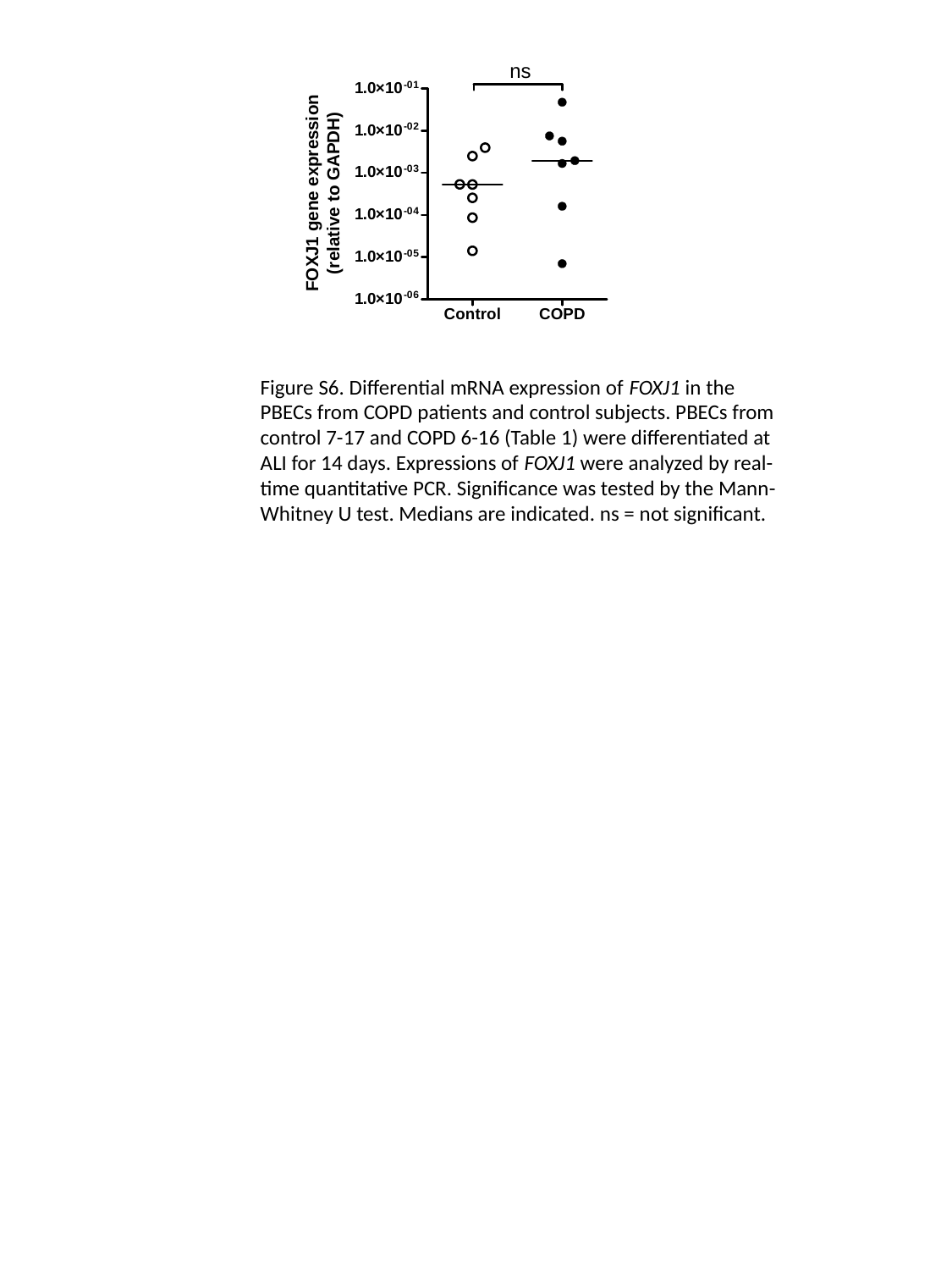

Figure S6. Differential mRNA expression of FOXJ1 in the PBECs from COPD patients and control subjects. PBECs from control 7-17 and COPD 6-16 (Table 1) were differentiated at ALI for 14 days. Expressions of FOXJ1 were analyzed by real-time quantitative PCR. Significance was tested by the Mann-Whitney U test. Medians are indicated. ns = not significant.
